# miR-217 inhibits triple-negative breast cancer cell growth, migration, and invasion through targeting KLF5

**DOI:** 10.1371/journal.pone.0176395

**Published:** 2017-04-24

**Authors:** Wenhui Zhou, Fangfang Song, Qiuju Wu, Rong Liu, Lulu Wang, Cuicui Liu, You Peng, Shuqin Mao, Jing Feng, Ceshi Chen

**Affiliations:** 1 Third Clinical College, Southern Medical University, Guangdong Province, Guangzhou, China; 2 Department of Laboratory Medicine & Central Laboratory, Southern Medical University Affiliated Fengxian Hospital, Shanghai, China; 3 Key Laboratory of Animal Models and Human Disease Mechanisms of Chinese Academy of Sciences & Kunming Institute of Zoology, Chinese Academy of Sciences, Yunnan Province, Kunming, China; 4 Department of Laboratory Medicine & Central Laboratory, Jinzhou Medical University Affiliated Fengxian Hospital, Shanghai, China; 5 Hubei University of Medicine Affiliated Taihe Hospital, Hubei Province, Shiyan, China; University of South Alabama Mitchell Cancer Institute, UNITED STATES

## Abstract

Triple negative breast cancer (TNBC) is one of the most aggressive breast cancers without effective targeted therapies. Numerous studies have implied that KLF5 plays an important roles in TNBC. How is KLF5 regulated by microRNAs has not been well studied. Here, we demonstrated that miR-217 down-regulates the expression of KLF5 and KLF5’s downstream target gene FGF-BP and Cyclin D1 in TNBC cell lines HCC1806 and HCC1937. Consequently, miR-217 suppresses TNBC cell growth, migration, and invasion. MiR-217 suppresses TNBC, at least partially, through down-regulating the KLF5 expression. These results suggest that the miR-217-KLF5 axis might serve as a potential target for treatment of TNBC.

## Introduction

Breast cancer is still the main cause of female cancer-related death in United States [1]. Triple-negative breast cancer (TNBC), which lacks expression of estrogen receptor α (ERα) and progesterone receptor (PR) and human epidermal growth factor receptor 2 (HER2) gene, accounts for approximately 15% of breast cancers [2]. TNBC is more aggressive, has higher rates of relapse and shorter overall survival than other subtypes of breast cancers. The median survival of women with metastatic TNBC is less than 12 months [3]. Up to now, there are still no effective targeted therapies for TNBC. Therefore, it’s urgent to identify effective therapeutic targets for TNBC.

Krüppel-like factor 5 (KLF5), a member of KLF family, is highly expressed in the epithelial crypt cells of the gastrointestinal tract and involved in diverse cellular functions [4–6]. In breast cancer, KLF5 plays an important role in tumorigenesis and high expression level of KLF5 is positively correlated with poor survival rate of breast cancer patients [7, 8]. We previously found that KLF5 promotes breast cancer cell proliferation, migration and invasion by upregulating the expression of FGF-BP, mPGES1, and TNFAIP2 and down-regulating the expression of p27 [9–12]. KLF5 depletion leads to breast cancer cell apoptosis and suppresses xenograft tumor growth *in vivo* [13, 14]. Importantly, pharmacological inhibition of KLF5 by mifepristone leads to suppression of TNBC stem cells [15]. In conclusion, KLF5 is a potential therapeutic target for TNBC.

MicroRNAs are a class of endogenous 21-23-nucleotide non-coding RNAs, usually function through inhibiting translation or inducing degradation of target mRNAs via binding to the specific sites on the 3’UTR of target mRNAs and are involved in diverse cellular functions [16, 17]. Numerous studies demonstrated that microRNAs could serve as either tumor suppressor or oncogene depending on the target genes’ functions [18]. Both microRNAs and anti-microRNA constructs are now under investigation as potential therapeutic agents for cancer. At present, the first microRNA mimics clinical trial is in progress [19].

MiR-217 was first illustrated to function as a potential tumor suppressor by targeting KRAS in pancreatic ductal adenocarcinoma [20–22]. Additionally, miR-217 induces endothelial cell senescence by targeting SirT1 [23]. MiR-217 inhibits clear cell renal cell carcinoma, hepatocellular carcinoma, gastric cancer and glioma by targeting E2F3, EZH2 and Runx2, respectively [24–27]. In contrast, miR-217 was proposed to be an oncogene by targeting PTEN, DACH1, PPARγ co-activator 1-α (PGC1-α) and enhances the germinal center reaction [28–31]. Based on these reports, it is attempting to speculate that miR-217 has context-dependent functions in carcinogenesis.

In this study, we found that miR-217 downregulates the KLF5 expression via binding to its 3’UTR and suppresses TNBC cell growth, migration, and invasion. Furthermore, we showed that miR-217 inhibits TNBC, at least in part, through targeting KLF5. Our findings support that miR-217 functions as a tumor suppressor in TNBC.

## Methods and materials

### Cell culture and western blot

HCC1937, HCC1806 and HEK293T were obtained from ATCC (American Type Culture Collection, Manassas, VA, USA). HCC1937 and HCC1806 cells were cultured in RPMI 1640 medium (Gibco, Carlsbad, CA, USA) supplemented with 10% FBS (fetal bovine serum, Gibco), at 37℃ with 5% CO_2_, and HEK293T was maintained in DMEM medium (Gibco) with 5% FBS. Western blot and the anti-KLF5 antibody have been described in our previous study [32]. The anti-FGF-BP (MAB1593) antibody was purchased from R&D Systems (Minneapolis, MN, USA). The anti-β-actin (A5441) antibody was purchased from Sigma-Aldrich (St Louis, MO, USA). The anti-DACH1 (10914–1) antibody was purchased from Protein Tech (Chicago, IL, USA). The anti-pTEN (#9559) antibody was purchased from Cell Signaling (Boston, MA, USA).

### Cell infection

4×10^5^ HCC1937 and 5×10^5^ HCC1806 cells were seeded in a 6 cm cell culture dishes. On the next day, 30 μl of lentiviral Lv-miR-217 or control (1×10^9^/ ml, Shanghai GenePharma Co., Ltd) together with polybrene (10 μg/ml final concentration) were added to the cells. The media were changed 48 hours later. The cells were treated with puromycin (1 μg/ml) to establish miR-217 stable over-expression cell populations.

### Cell viability assay

The cell viability was measured by SRB (sulforhodamine B) assays. Briefly, cells were seeded in 48-well plates at a density of 8.0×10^3^ cells per well. The cells were fixed with 10% TCA at 4℃ for 60 minutes at indicated time, followed by incubating with 0.4% SRB (W/V) solution in 1% acetic acid for 5 minutes at room temperature. At last, the SRB was dissolved with 10 mM unbuffered Tris base, and the absorbance was measured at a single wavelength of 490–530 nm on plate reader (Bio Tek, VT, USA). All experiments were performed in triplicates at least twice independently.

### Wound healing, migration, and matrigel invasion assays

The wound healing assay was performed in 6-well plates. 1.2×10^6^ HCC1806 cells or 1×10^6^ HCC1937 cells were seeded in each well of a 6-well plate. Twenty four hours after cell plating, wounds were made using a pipette tip, and the wounds were recorded under a microscope every 4 hours. The cell migration and matrigel invasion assays were evaluated using 24-well chemotaxis chambers (Corning cell culture inserts, 8 μm pore size) and chemotaxis matrigel chambers (BD BIOCOAT Matrigel chambers, 8 μm pore size) according to published protocol [10].

### Real-time PCR

Total RNA was extracted using Trizol reagent (Invitrogen, Carlsbad, CA, USA). Reverse transcription was performed using the TaqMan PCR MicroRNA Reverse Transcription Kit (Applied Biosystems, Austin, TX, USA). For quantitative PCR, SYBR PCR Master Mix (Applied Biosystems) was used to quantify the expression level of RNA on a 7900HT Fast Real-Time PCR System (Applied Biosystems). The miR-217 expression level was detected using a special Bulge-Loop^TM^ miRNAs qPCR Primer Set (Guangzhou RiboBio Co., LTD). U6 was used as the endogenous loading control.

### Dual luciferase assays

The 1652-bp KLF5 3’UTR has been cloned into the pMIR-REPORT™ miRNA Expression Reporter Vector [15]. The mutants were generated by PCR using four special primers: Wile type, forward primer: *5’-TGTGGTAAGGTACCTCTCAACATTAC-3’* and reverse primer: *5’-GCCCTTTGGTTAACAGCATCAGCATC-3’*; mut1: forward primer *5’-ATGACAATGTTGCATTTATG****TACGTCA****TTCAAGTACCAAAACGTTGA-3’* and reverse primer *5’-TCAACGTTTTGGTACTTGAA****TGACGTA****CATAAATGCAACATTGTCAT-3’*; mut2: forward primer *5’-ACCAAAACGTTGAATTGATG****TACGTCA****TTTCATATATCGAGATGTTC-3’* and reverse primer *5’-GA ACATCTCGATATATGAAA****TGACGTA****CATCAATTCAACGTTTTGGT-3’*. For dual luciferase reporter assay, 8×10^3^ HEK293T cells per well was seeded in 12-well plates. Eighteen hours after plating, the cells were transfected with wild type or mutated KLF5 3’UTR luciferase reporter constructs (0.6 μg per well) and an internal control pCMV-Renilla (0.2 μg per well) along with miR-217 mimics or the negative control using Lipofectamine 2000 (Invitrogen). The luciferase activities were measured using the dual luciferase reporter assay system (Promega, Madison, WI, USA) 48 hour after transfection.

### Statistical analysis

All of the results were shown as Mean±SD, and statistical analysis were performed by Student’s t-test using the SPSS program (version 12.0) (SPSS, Chicago, IL, USA). P<0.05 was defined to be statistically significant (*, P<0.05, **, P<0.01, ***, P<0.001).

## Results

### miR-217 directly targets KLF5

**Fig 1:**
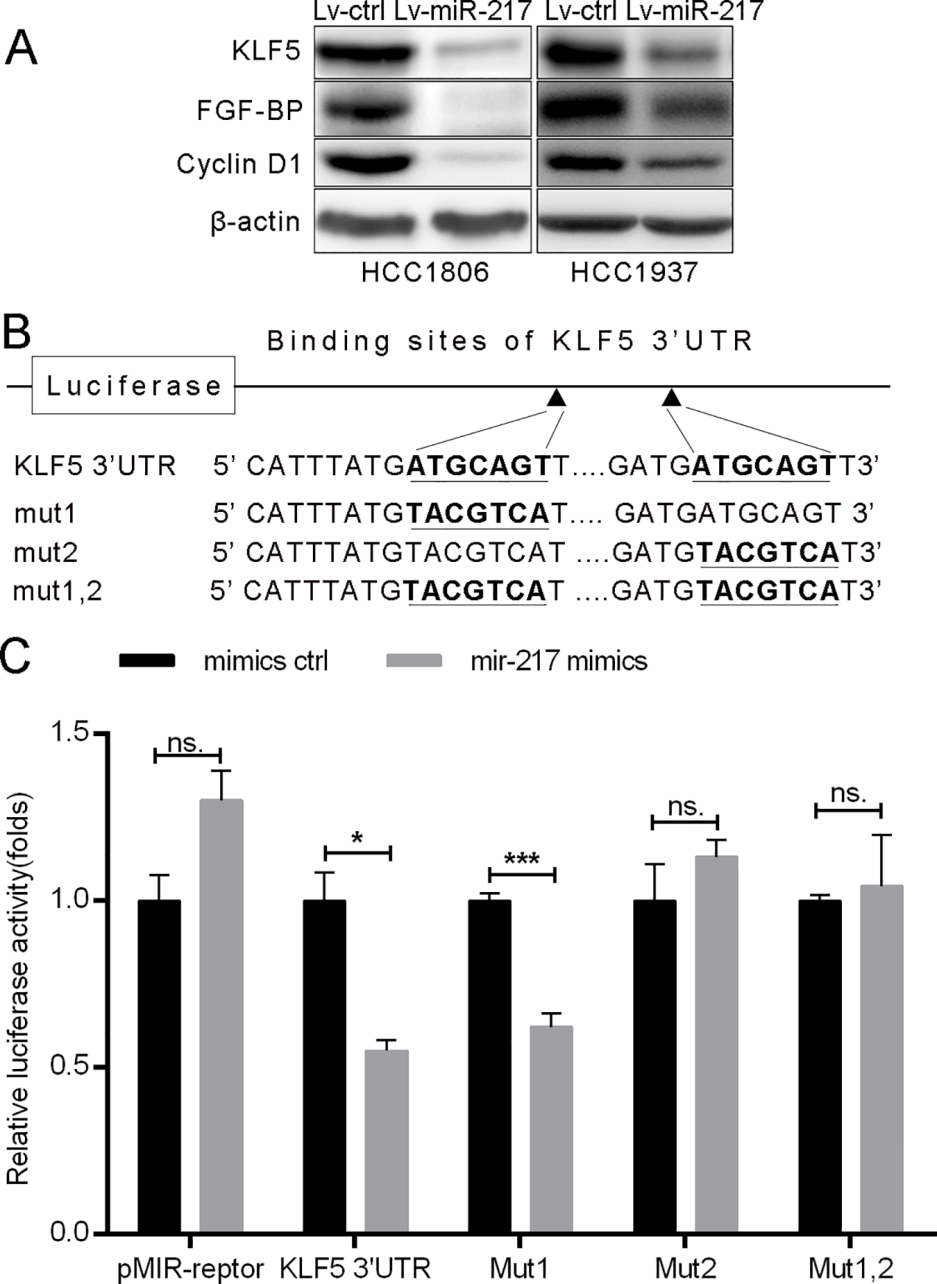
miR-217 targets KLF5 by binding to its 3’UTR. **A.** miR-217 decreased the KLF5, FGF-BP and Cyclin D1 protein levels in HCC1806 and HCC1937 TNBC cells. KLF5, FGF-BP and Cyclin D1 protein levels were detected by using WB. β-actin was used as the loading control. **B.** The putative wild type binding sites of miR-217 on KLF5 3’UTR and its mutants. **C.** miR-217 mimics significantly inhibits the KLF5 3’UTR luciferase reporter activity through the second putative binding site. HEK293T cells were transfected with miR-217 mimics and pMIR-KLF5 3’-UTR or miR-217 binding sites mutated pMIR-KLF5 3’-UTR reporters (mut1, mut2 or mut1,2) together with the pCMV-Renilla control.

To identify target genes of miR-217, we employed online microRNA target prediction softwares, including Targetscan, miRwalk and miRTarBase, and identified KLF5 as a potential miR-217 target. We then tested whether miR-217 indeed inhibits the KLF5 expression in TNBC cell lines, HCC937 and HCC1806. As shown in Fig 1A, miR-217 suppressed KLF5 and its direct downstream targets FGF-BP and Cyclin D1 protein expression in both cell lines. We then analyzed the 3’UTR of KLF5 and found two possible binding sites for miR-217 (Fig 1B). To further test whether miR-217 inhibits the KLF5 expression through putative binding sites at KLF5 3’UTR, we performed dual luciferase reporter assay. As expected, miR-217 significantly suppressed the luciferase activity when the luciferase reporter gene linked with wild type KLF5 3’-UTR; however, the luciferase activity of the second, but not the first, miR-217 binding site mutated reporters was not inhibited by miR-217 (Fig 1C). These results implicated that miR-217 targets KLF5 through binding to the second putative site on the KLF5 3’UTR.

### miR-217 inhibits TNBC cell growth and migration

**Fig 2:**
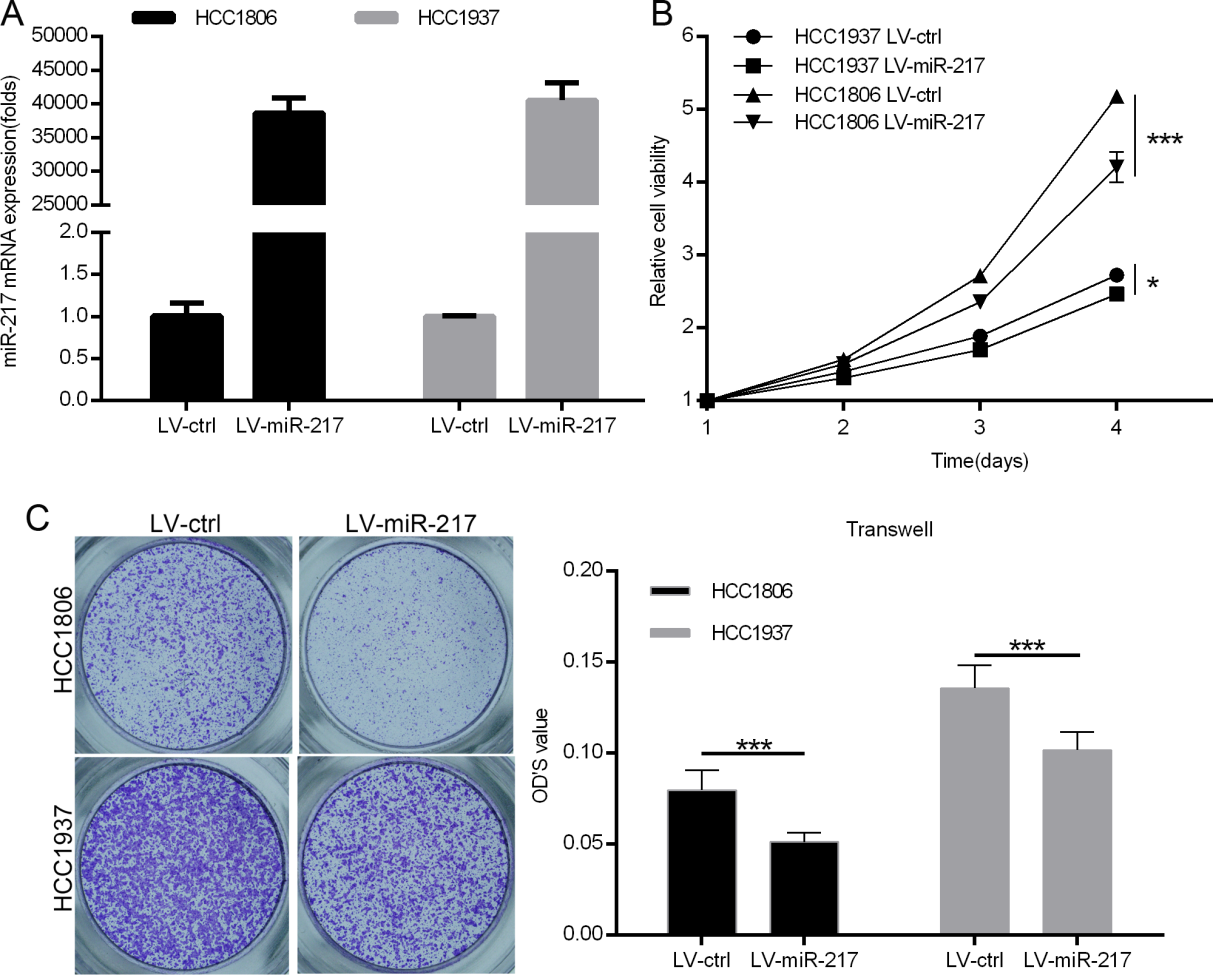
miR-217 inhibits TNBC cell growth and migration. **A.** miR-217 was stably overexpressed in HCC1806 and HCC1937 cell lines. The miR-217 level was measured by quantitative PCR. **B.** miR-217 suppressed TNBC cell growth. The growth of HCC1806 and HCC1937 was measured using the SRB assay. **C.** miR-217 suppressed TNBC cell migration. miR-217 overexpression or vector control HCC1806 and HCC1937 cells were plated in chemotaxis chambers for 24 or 8 hours, respectively, before being fixed for migration detection. Quantative results are shown in the right panel.

KLF5 is well established to promote TNBC cell proliferation and migration [10, 12, 15, 33, 34]. However, miR-217 has been reported to promote breast cancer cell proliferation [29, 30, 35]. To determine the functions of miR-217 in TNBC, we over-expressed miR-217 in HCC1937 and HCC1806 cells. The over-expression of miR217 was confirmed by qPCR (Fig 2A). As shown in Fig 2B, we found that miR-217 significantly suppressed cell growth, as determined by the SRB assay in both cell lines. We also investigated the effects of miR-217 on cell migration by the transwell migration assay and found that miR-217 significantly inhibited TNBC cell migration compared to the vector control (Fig 2C).

### miR-217 inhibits TNBC cell growth, migration, and invasion through KLF5

**Fig 3:**
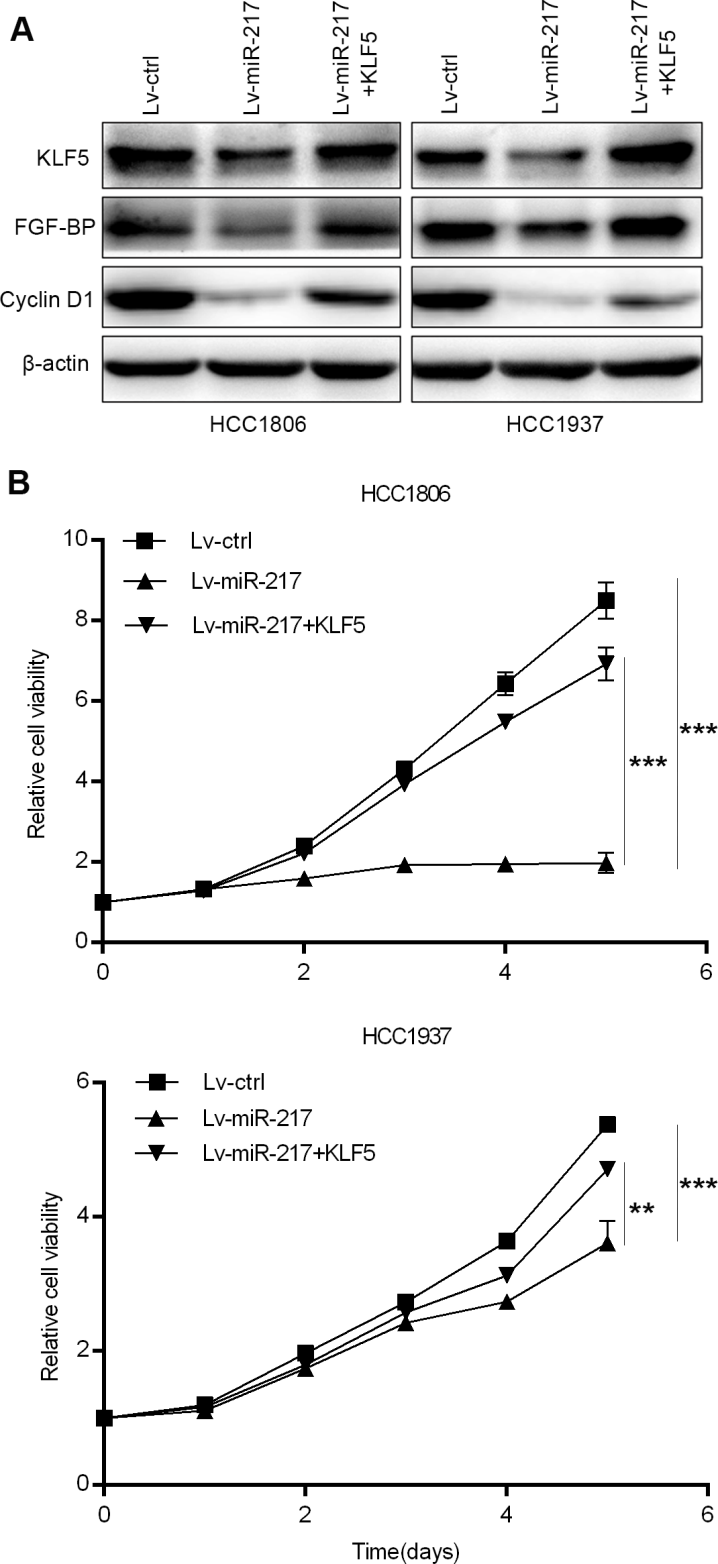
miR-217 suppresses TNBC cell growth through inhibiting KLF5. **A.** Ectopic over-expression of KLF5 in HCC1806 and HCC1937 cell lines restored the reduction of FGF-BP and Cyclin D1 expression caused by miR-217. The cells were transiently transfected with pBabe-KLF5 for 48 hours before WB. **B.** KLF5 overexpression significantly rescued miR-217-induced HCC1806 and HCC1937 cell growth inhibition. The cell growth was measured using the SRB assay.

**Fig 4:**
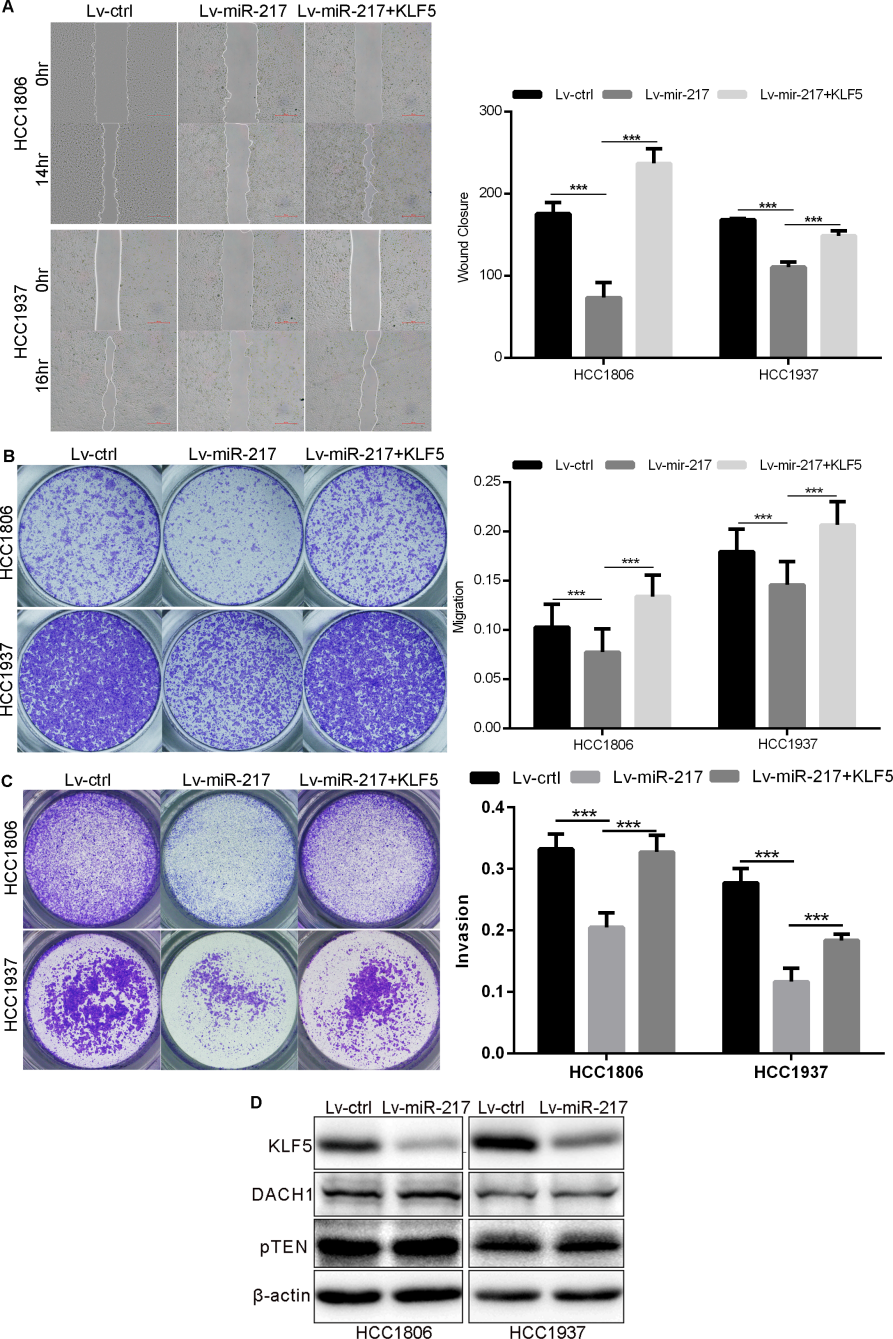
miR-217 inhibits TNBC cell migration and invasion through KLF5. **A.** KLF5 over-expression significantly rescued miR-217-induced cell migration inhibition, as measured by the wound healing assay. Quantative results are shown in the right panel. **B.** KLF5 over-expression significantly rescued miR-217-induced cell migration inhibition, as measured by the transwell migration assay. **C.** KLF5 over-expression significantly rescued miR-217-induced cell invasion inhibition, as measured by the matrigel transwell invasion assay. **D.** miR-217 did not decrease the expression of DACH1 and pTEN in HCC1806 and HCC1937 cells, as measured by WB.

To test whether miR-217 regulates TNBC growth, migration, and invasion via targeting KLF5, we ectopically over-expressed KLF5 in miR-217 transfected HCC1937 and HCC1806 cells. As shown in Figs 3 and 4, KLF5 restoration in both HCC1937 and HCC1806 cells almost completely rescued miR-217-caused FGF-BP and Cyclin D1 decrease (Fig 3A), inhibition of cell growth (Fig 3B), migration (Fig 4A and 4B), and invasion (Fig 4C).

Additionally, we tested whether miR-217 targets DACH1 and pTEN in HCC1937 and HCC1806 cells. As shown in Fig 4D, although miRNA-217 significantly decreased the KLF5 protein levels in both cell lines, the protein levels of DACH1 and pTEN were not decreased. These results suggest that miR-217 predominately functions through KLF5 in these two TNBC cell lines.

## Discussion

The expression of KLF5 is correlated with different hormone status in different breast cancer subtypes [8]. Accumulated evidence suggests that KLF5 is an oncogene in TNBC [7, 10, 12–15, 36]. We previously have reported KLF5 promotes TNBC cell proliferation, survival, migration and invasion via regulating a variety of target genes; however, how KLF5 is regulated in breast cancer needs to be further elucidated. To date, only miR-153 has been reported to target KLF5 in TNBC [15]. In this study, we reported that the protein level of KLF5 is downregulated by miR-217 in TNBC cells. MiR-217 directly binds to a putative sequence (968–974 nt) at the *KLF5* gene 3’UTR although there are two candidate sequences. It is possible that the flanking sequences determine the binding specificity. Finally, we demonstrated that miR-217 inhibits TNBC cell growth, migration, and invasion, at least partially through KLF5. The miR-217-KLF5 axis might serve as a new target for TNBC therapy.

A microRNA could function as either a tumor suppressor or promoter in different cancers in a context-dependent manner because it targets numerous different target genes. Although miR-217 is overexpressed in aggressive human B-cell lymphomas and promotes mature B-cell lymphomagenesis [28], most studies suggest that miR-217 functions as a tumor suppressor in a variety of cancers. In pancreatic ductal adenocarcinoma, miR-217 inhibits tumor cell growth by targeting KRAS and SIRT1 [22, 37]. Additionally, downregulation of miR-217 in Ph(+) leukemia cells causes resistance to ABL tyrosine kinase inhibitors [38]. Furthermore, miR-217 was reported to inhibit invasion of hepatocellular carcinoma cells through targeting E2F3 [26]. In esophageal squamous cell carcinoma cells, miR-217 inhibits proliferation, migration, and invasion by targeting long noncoding RNA MALAT1 and kallikrein 7 (KLK7) [39]. Interestingly, miR-217 is down-regulated by cigarette smoke [40]. miR-217 was suggested to be a tumor suppressor by targeting Wnt5a in osteosarcoma and targeting IGF1R in ovarian cancer [41, 42].

In breast cancer, Zhang reported that miR-217 promotes MCF-7 and MDA-MB-231 cell proliferation via targeting DACH1 [30]. Overexpression of miR-217 induces drug resistance and invasion of MCF-7 and SKBR-3 cells through activating AKT by downregulation of PTEN [35]. Furthermore, downregulation of miR-217 in MCF7 and MDA-MB-231 cells inhibits cell proliferation through targeting PGC-1α and DACH1 [29, 30]. These studies suggest an oncogenic role of miR-217 in breast cancer. However, we found miR-217 suppressed cell growth, migration, and invasion via targeting KLF5 in both HCC1937 and HCC1806 cell lines. Coincidently, KLF5 is lowly expressed in MCF-7, MDA-MB-231, and SKBR3 but highly expressed in HCC1937 and HCC1806 ([33] and unpublished results). Additionally, miR-217 did not inhibit the expression of DACH1 and pTEN in both HCC1937 and HCC1806 cell lines. Therefore, miR-217 may has a context-dependent roles in a cell line specific manner in breast cancer.

We recently reported that mifepristone inhibits KLF5 expression and cancer stem cells in basal TNBC [15]. Additionally, Curcumin inhibits KLF5 expression through YAP/TAZ in bladder cancer cell lines [43]. ML264 inhibits KLF5 expression in colorectal cancer cell lines [44, 45]. microRNA mimics could be used as drugs for cancer treatment [19]. Therefore, we think that KLF5 pharmacological inhibition and miR-217 may be used for TNBC treatment in the future.

In summary, we conclude that miR-217 inhibits TNBC cell growth, migration, and invasion *in vitro* through targeting the KLF5 transcription factor. The miR-217-KLF5 axis may be used for TNBC therapy.
