# Correction: miR-217 inhibits triple-negative breast cancer cell growth, migration, and invasion through targeting KLF5

**DOI:** 10.1371/journal.pone.0354115

**Published:** 2026-07-20

**Authors:** The PLOS One Editors

Following the publication of this article [1], concerns were raised regarding results presented in Fig 4 and the statistical approach described in the article. Specifically,

- The Fig 4A HCC1806 0h Lv-miR-217 panel appears similar to the Fig 4A HCC1937 0hr Lv-miR-217 panel.
- In Fig 4D, the PTEN signal observed in the HCC1937 panels is unexpected, considering the HCC1937 cell line is characterized by a homozygous deletion of the PTEN gene, raising concerns about cell line contamination.
- The Methods and materials section only reports the use of Student’s t-test and does not clarify the statistical approach used for multivariate analysis; however, multiple figures in this article appear to present multivariate results.

Corresponding author CC stated that the Fig 4A HCC1937 0hr Lv-miR-217 panel is incorrect and provided an updated Fig 4 in which the incorrect Fig 4A HCC1937 0hr Lv-miR-217 panel has been replaced with the correct panel from the original experimental results. The triplicate image data underlying Fig 4 are available in S1 File.

Regarding the cell line concerns, corresponding author CC stated that after re-examining the original western blot data they agree that the PTEN signal shown for HCC1937 in Fig 4D should not be interpreted as evidence of bona fide PTEN protein expression. They state that the HCC1937 cell line used in this study was obtained from ATCC and they provided details from the STR profiling report obtained for this study (S2 File). Instead, corresponding author CC believes that the PTEN band shown for HCC1937 in Fig 4D was most likely a non-specific antibody signal, possibly enhanced by increased sample loading and prolonged exposure, and mistakenly interpreted as low-level PTEN expression in HCC1937 cells. Following this re-examination, the PTEN results have been removed from the updated Fig 4D, and in addition to updates to the figure legend for Fig 4D, the sections listed below have been updated.

In the ‘miR-217 inhibits TNBC cell growth, migration, and invasion through KLF5’ subsection of the Results section, the second paragraph is updated to:

Additionally, we tested whether miR-217 targets DACH1 in HCC1937 and HCC1806 cells. As shown in Fig 4D, although miR-217 significantly decreased the KLF5 protein levels in both cell lines, the protein level of DACH1 was not decreased. These results are consistent with the interpretation that miR-217 functions predominantly through KLF5 in these two TNBC cell lines.

In the Discussion section, the seventh and eighth sentences of the third paragraph are updated to:

Additionally, miR-217 did not inhibit the expression of DACH1 in both HCC1937 and HCC1806 cell lines. Therefore, miR-217 may have a context-dependent role in a cell line specific manner in breast cancer.

Regarding the statistical concerns, the authors state that the statistical approach reported in the Methods and materials section is incomplete and they explained that one-way and two-way ANOVAs were performed (as appropriate), followed by Student’s t-test for post-hoc comparisons.

Contrary to the declaration in the Data Availability statement, the original raw data files supporting the article’s results were not provided with the article. The authors have provided the original individual-level data, which is available in S3 File.

The data in S3 File and the newly reported statistical approach and updated findings were assessed by a Statistical Advisor, who raised several additional concerns:

1. Figs 2B and 3B: Further clarification and reasoning is required to explain why different statistical tests were used when the datasets underlying these figures appear similar.
2. Fig 2C: it is unclear how the samples subjected to paired t-tests were matched.
3. Fig 3B: the article does not comment on the observation that the comparison “Lv-miR-217+KLF5 vs. Lv-ctrl” appears to be significant.

Regarding the first point above, the authors agreed with the Statistical Advisor’s concern about different statistical approaches being employed for the results in Fig 2B (single factor design) versus Fig 3B (two-factor design), and they updated the analyses for both figures using two-way ANOVA (group * time; no matching and no repeated measures, as the measurement for each day was obtained from different wells). The authors report that the updated results show that Lv-miR-217 significantly suppresses growth of HCC1806 and HCC1937 cells, and KLF5 re-expression significantly restores proliferative capacity, which is consistent with the overall conclusions of the published article.

Regarding the second point above, the authors clarified that the pairing for the paired t-test reported for Fig 2C was done by independent experimental runs (batch), stating that they observed batch-to-batch shifts in overall migration/invasion read-outs. The authors clarify that they treated each independent experiment as a matched block and compared groups within the same experiment.

Regarding the third point above, the authors agree with the Statistical Advisor’s observation but state that they interpret these results as indicating that KLF5 rescues partially, but not necessarily completely reverses, the miR-217–mediated phenotype under the tested experimental conditions. They state that this could reflect variability in KLF5 re-expression levels across cells or the possibility that miR-217 influences proliferation through additional targets beyond KLF5, but that this observation does not conflict with the article’s main conclusion that KLF5 is a functional downstream mediator and instead the authors state that the observation suggests that the rescue may be incomplete.

The Statistical Advisor assessed the authors’ responses and concluded that their concerns were sufficiently addressed.

In addition to the above concerns:

- The text “pMIR-reptor” in the x-axis label for Fig 1C is incorrect, and has been updated to “pMIR-REPORT” in the updated Fig 1 provided with this notice.
- The Fig 4D HCC1937 KLF5 panel of this article [1] appears similar to the Fig 4A GATA6 panel of [2] published at a later date by a different, unaffiliated author group. PLOS has no concerns about the Fig 4D HCC1937 KLF5 panel of this article [1].

**Fig 1:**
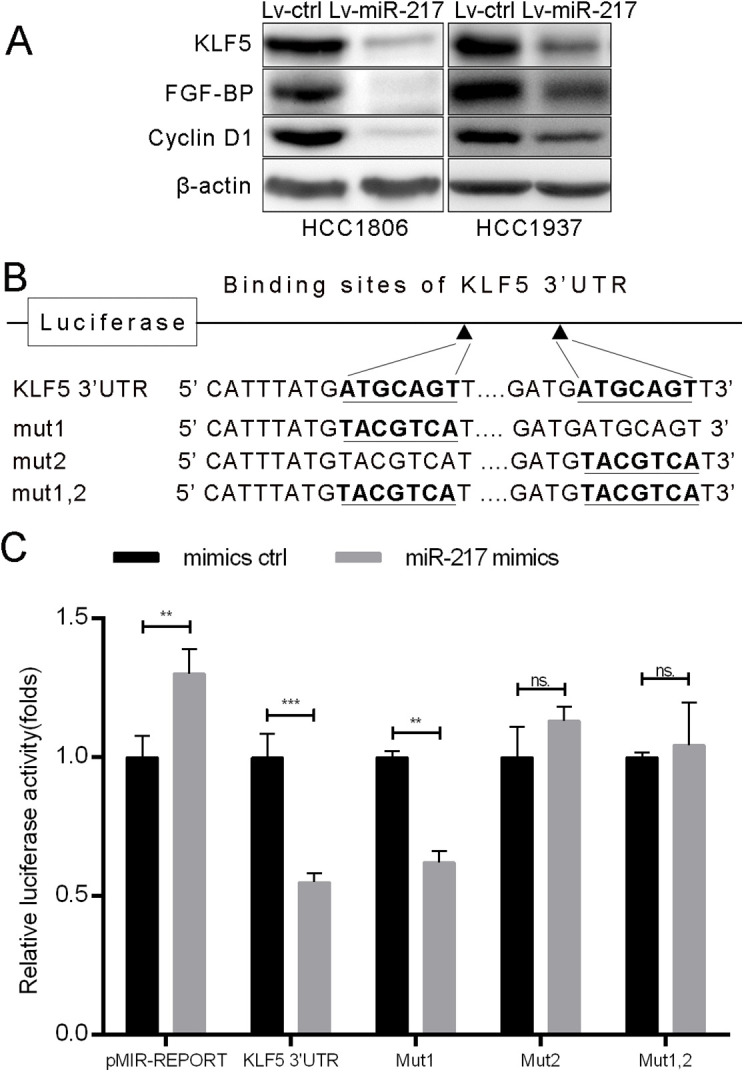
miR-217 targets KLF5 by binding to its 3’UTR. **A.** miR-217 decreased the KLF5, FGF-BP and Cyclin D1 protein levels in HCC1806 and HCC1937 TNBC cells. KLF5, FGF-BP and Cyclin D1 protein levels were detected by using WB. β-actin was used as the loading control. **B.** The putative wild type binding sites of miR-217 on KLF5 3’UTR and its mutants. **C.** miR-217 mimics significantly inhibits the KLF5 3’UTR luciferase reporter activity through the second putative binding site. HEK293T cells were transfected with miR-217 mimics and pMIR-KLF5 3’-UTR or miR-217 binding sites mutated pMIR-KLF5 3’-UTR reporters (mut1, mut2 or mut1,2) together with the pCMV-Renilla control. Data are represented as mean ± SD. *p < 0.05, **p < 0.01, and ***p < 0.001 by twoway ANOVA. ns, not significant. n = 3 biological replicates.

**Fig 2:**
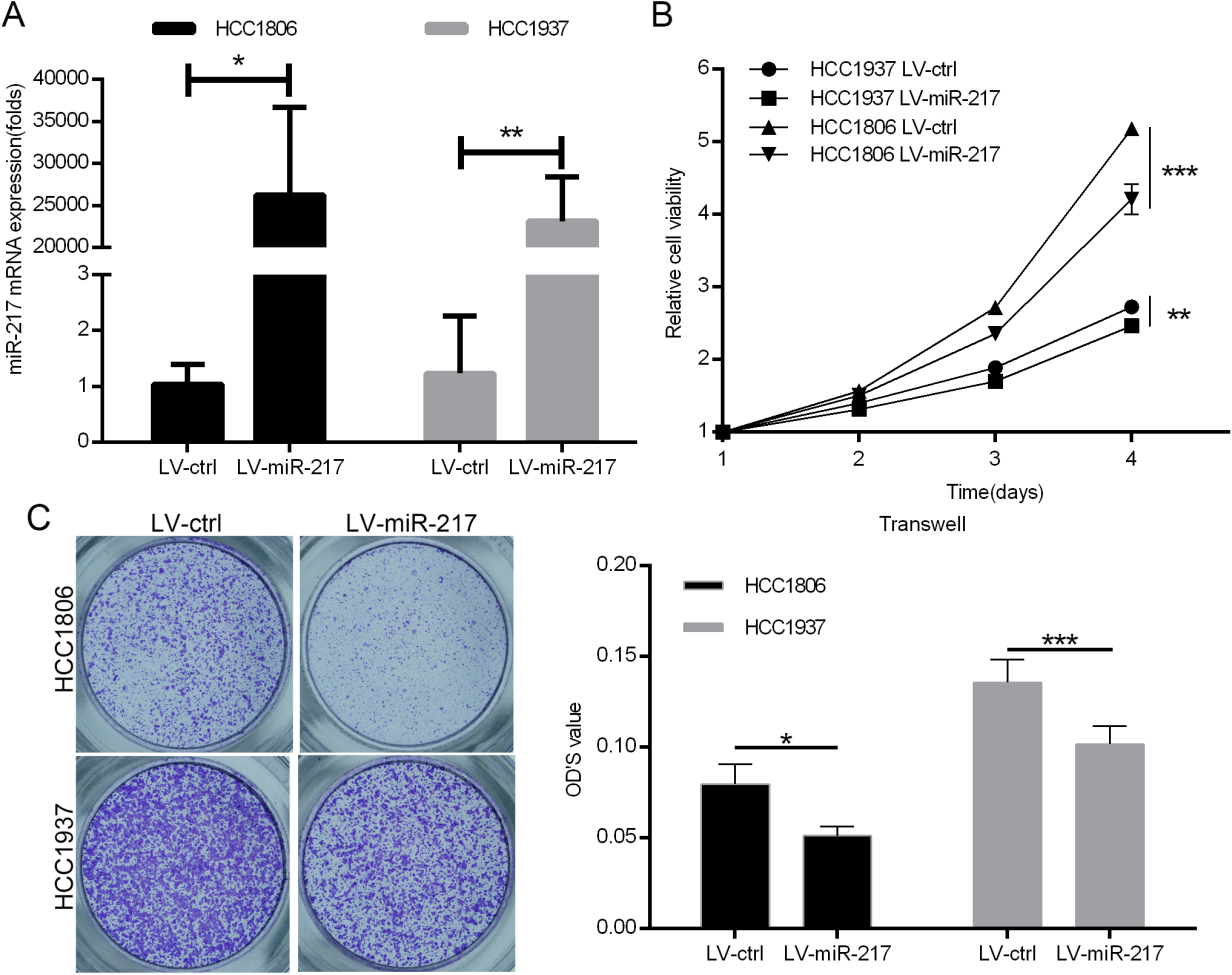
miR-217 inhibits TNBC cell growth and migration. **A**. miR-217 was stably overexpressed in HCC1806 and HCC1937 cell lines. The miR-217 level was measured by quantitative PCR. Data are represented as mean ± SD. *p < 0.05, **p < 0.01, and ***p < 0.001 by unpaired Student's t test. ns, not significant. n = 3 biological replicates. **B.** miR-217 suppressed TNBC cell growth. The growth of HCC1806 and HCC1937 was measured using the SRB assay. Data are represented as mean ± SD. *p < 0.05, **p < 0.01, and ***p < 0.001 by twoway ANOVA. ns, not significant. n = 3 biological replicates. **C.** miR-217 suppressed TNBC cell migration. miR-217 overexpression or vector control HCC1806 and HCC1937 cells were plated in chemotaxis chambers for 24 or 8 hours, respectively, before being fixed for migration detection. Quantative results are shown in the right panel. Data are represented as mean ± SD. *p < 0.05, **p < 0.01, and ***p < 0.001 by paired Student's t test. ns, not significant. n = 3 biological replicates.

**Fig 3:**
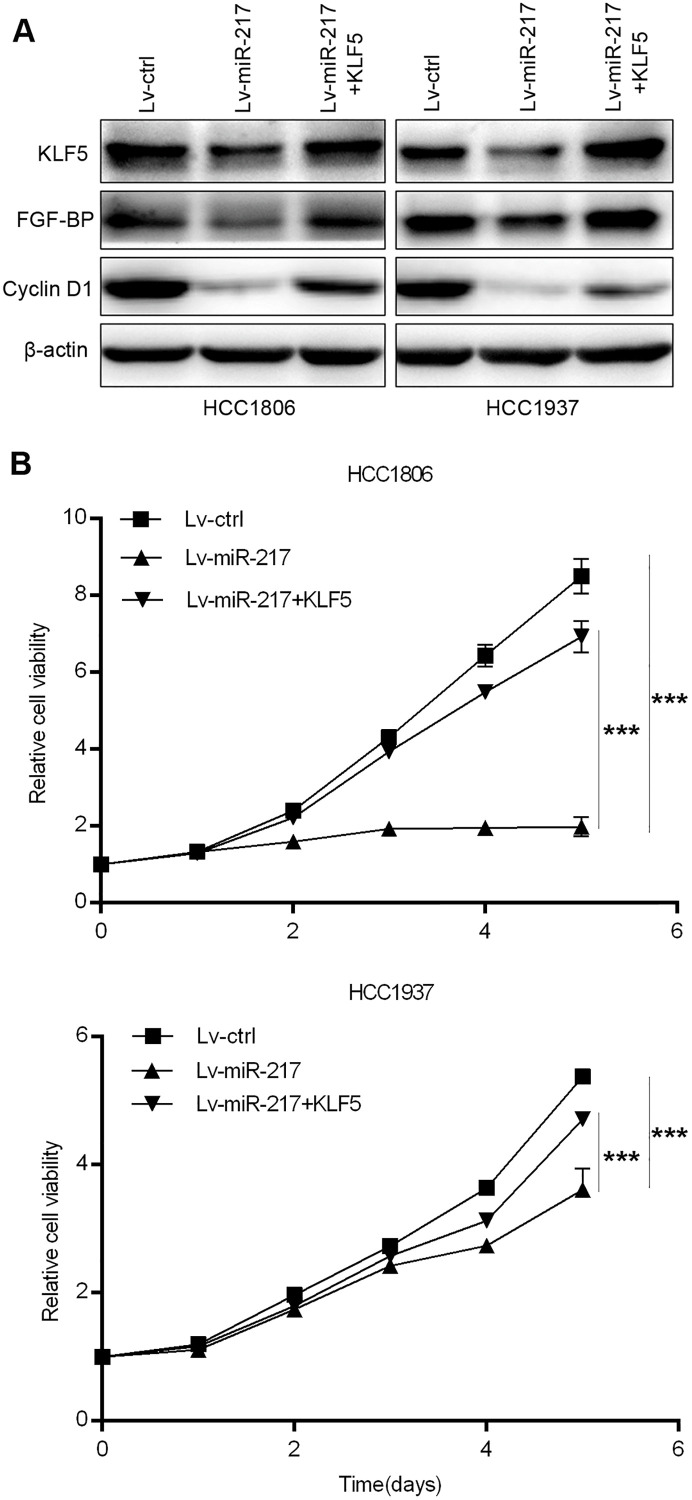
miR-217 suppresses TNBC cell growth through inhibiting KLF5. **A**. Ectopic over-expression of KLF5 in HCC1806 and HCC1937 cell lines restored the reduction of FGF-BP and Cyclin D1 expression caused by miR-217. The cells were transiently transfected with pBabe-KLF5 for 48 hours before WB. **B.** KLF5 overexpression significantly rescued miR-217-induced HCC1806 and HCC1937 cell growth inhibition. The cell growth was measured using the SRB assay. Data are represented as mean ± SD. *p < 0.05, **p < 0.01, and ***p < 0.001 by twoway ANOVA. ns, not significant. n = 3 biological replicates.

**Fig 4:**
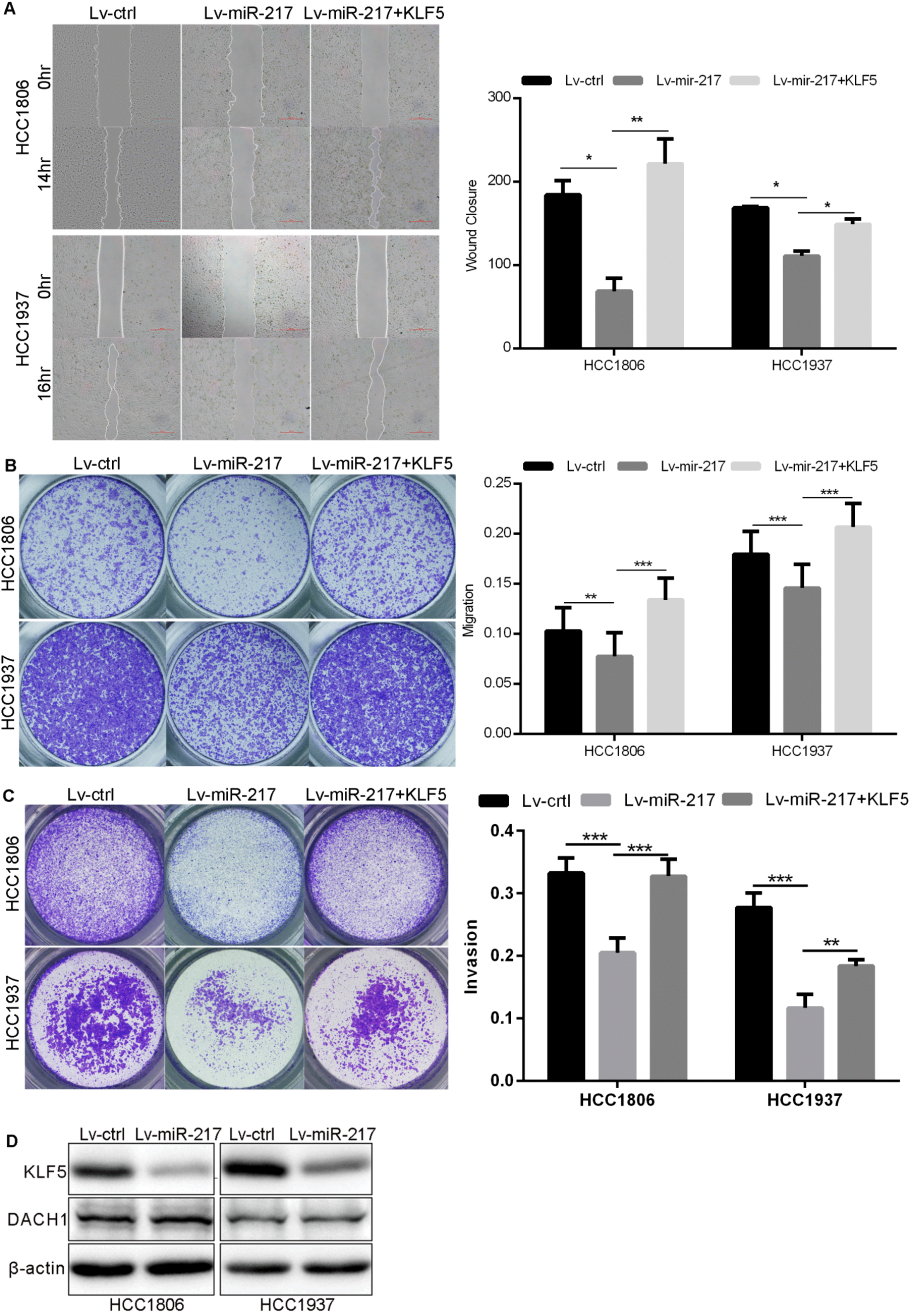
miR-217 inhibits TNBC cell migration and invasion through KLF5. **A**. KLF5 over-expression significantly rescued miR-217-induced cell migration inhibition, as measured by the wound healing assay. Quantative results are shown in the right panel. Data are represented as mean ± SD. *p < 0.05, **p < 0.01, and ***p < 0.001 by oneway ANOVA. ns, not significant. n = 3 biological replicates. **B.** KLF5 over-expression significantly rescued miR-217-induced cell migration inhibition, as measured by the transwell migration assay. Data are represented as mean ± SD. *p < 0.05, **p < 0.01, and ***p < 0.001 by oneway ANOVA. ns, not significant. n = 3 biological replicates. **C.** KLF5 over-expression significantly rescued miR-217-induced cell invasion inhibition, as measured by the Matrigel transwell invasion assay. Data are represented as mean ± SD. *p < 0.05, **p < 0.01, and ***p < 0.001 by oneway ANOVA. ns, not significant. n = 3 biological replicates. **D.** miR-217 decreased KLF5 expression but did not decrease the expression of DACH1 in HCC1806 and HCC1937 cells, as measured by WB.

## Supporting information

S1 File Triplicate image data underlying Fig 4. (PDF)

S2 File STR authentication report cell line HCC1937. (PDF)

S3 File Individual-level data and statistical analysis underlying Figs 1–4. (XLSX)
