# Supplementary figures and images for: Correction: miR-217 inhibits triple-negative breast cancer cell growth, migration, and invasion through targeting KLF5
Source: PLoS One. 2026 Jul 20;21(7):e0354115. doi: 10.1371/journal.pone.0354115 (PMC13384268; doi:10.1371/journal.pone.0354115)

Figure 4D. Original images

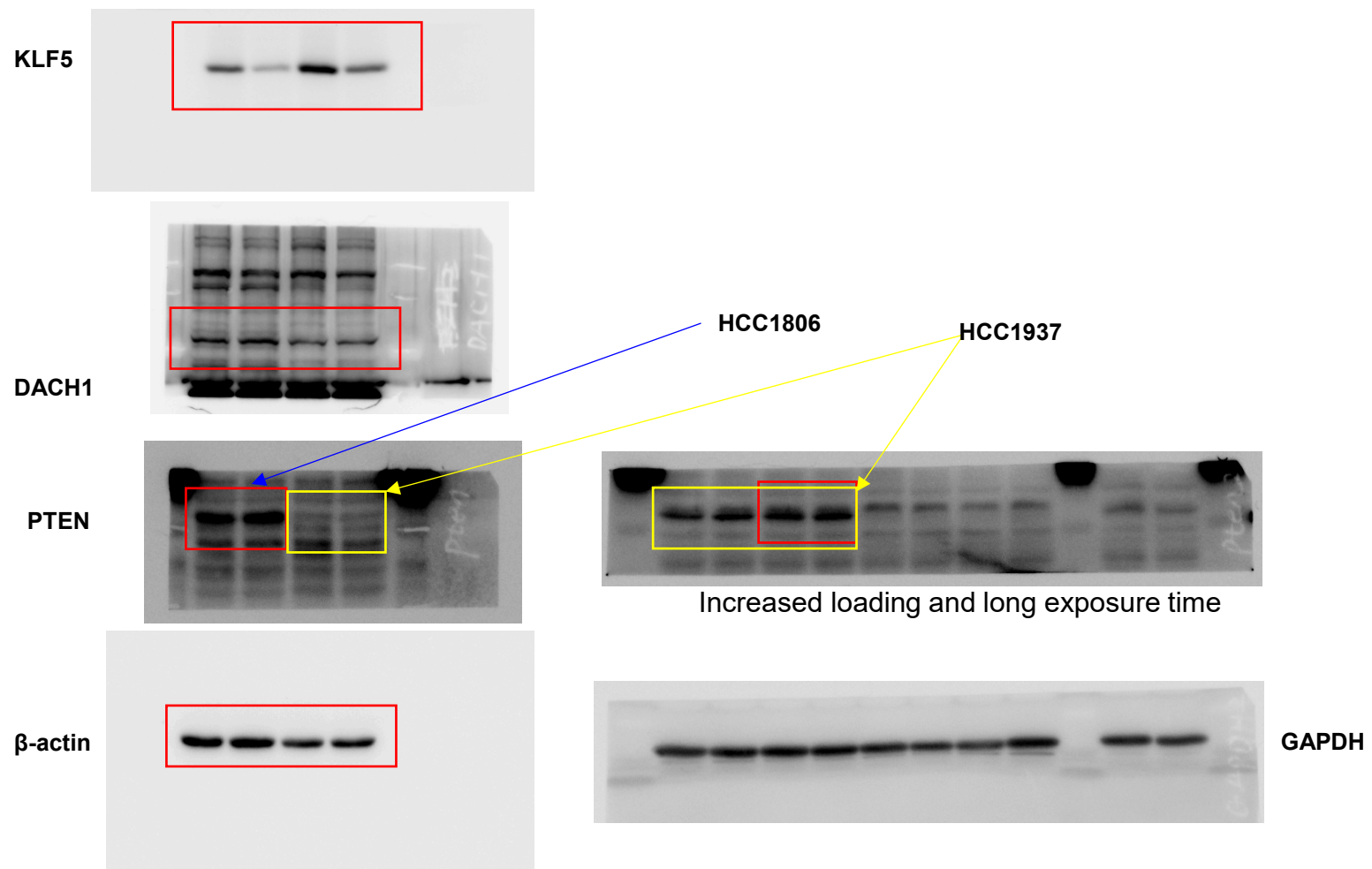

Supplement: S1 File — (PDF) [file pone.0354115.s001.pdf]
