# Supplementary material for: Correction: miR-217 inhibits triple-negative breast cancer cell growth, migration, and invasion through targeting KLF5
Source: PLoS One. 2026 Jul 20;21(7):e0354115. doi: 10.1371/journal.pone.0354115 (PMC13384268; doi:10.1371/journal.pone.0354115)
Supplement: S2 File — (PDF) [file pone.0354115.s002.pdf]

中国科学院昆明动物研究所昆明细胞库

人源细胞株 STR 检测报告单

细胞株名称：Hcc1937 人乳腺癌细胞

标本类型：细胞　　　　　　　　　　　送检单位：昆明动物研究所陈策实组

检测基因位点	Hcc1937（陈策实组）	Hcc1937（KCB）	Hcc1937（ATCC）
Amelogenin	X	X	X
D7S820	9,10	9,10	9,10
CSF1PO	12	12	12
TH01	6	6	6
D13S317	13	13	13
D16S539	13,14	13,14	13,14
vWA	16,17	16,17	16,17
TPOX	11	11	11
D5S818	12	12	12

结果： 送检 Hcc1937 细胞经和昆明细胞库及 ATCC 的 STR 基因位点比对，检测位点完全一致。系 Hcc1937 细胞。

报告日期：2015 年 7 月 23 日

地址：昆明市教场东路 32 号

本报告仅对该标本负责

电话：0871-65195375

Kunming Cell Bank, Kunming Institute of Zoology, Chinese Academy of Sciences

STR Authentication Report for Human Cell Line

Cell Line Name: HCC1937 human breast cancer cells
Specimen Type: Cells
Submitting Unit: Chen Ceshi Group, Kunming Institute of Zoology

STR Locus Tested	HCC1937 (Chen Ceshi Group)	HCC1937 (KCB)	HCC1937 (ATCC)
Amelogenin	X	X	X
D7S820	9,10	9,10	9,10
CSF1PO	12	12	12
TH01	6	6	6
D13S317	13	13	13
D16S539	13,14	13,14	13,14
vWA	16,17	16,17	16,17
TPOX	11	11	11
D5S818	12	12	12

Result:
The submitted HCC1937 cells were compared with the STR locus profiles of HCC1937 cells from the Kunming Cell Bank and ATCC. All tested loci were completely consistent. The submitted cells are confirmed to be HCC1937 cells.

Report Date: July 23, 2015
Address: No. 32 Jiaochang East Road, Kunming
Telephone: 0871-65195375

Note: This report applies only to the specimen tested.
